# Management of Oral Squamous Papilloma Using Annona squamosa (Custard Apple) Leaves: A Novel Case

**DOI:** 10.7759/cureus.34806

**Published:** 2023-02-09

**Authors:** Shikha Yadav

**Affiliations:** 1 Department of Dentistry, All India Institute of Medical Sciences, Mangalagiri, Mangalagiri, IND

**Keywords:** herbal medicine, complementary medicine, t/cam, annona squamosa leaves, human papilloma virus, oral papilloma, custard apple, squamous papilloma

## Abstract

This report presents the case of a 36-year-old male who was diagnosed with oral squamous papilloma in the palatal region. Excision was planned. The patient in the meantime chewed upon custard apple leaves and reported that the lesion prolapsed over the next few days. On examination, the site showed no signs of scarring or contracture and presented with healthy palatal tissue. The patient was prevented from requiring surgery, which would have shown a longer healing period with heavy reliance on an expensive resource base. This novel observation highlights the benefits of custard apple (*Annona squamosa) *leaves and warrants that its hepatoprotective, anticancer, antidiabetic, antioxidant, antibacterial, anti-obesity, and lipid-lowering properties are studied in an astute scientific setup with a well-drawn-out research plan.

## Introduction

Oral squamous papilloma (OSP) is a benign tumour seen in all age groups, more commonly in individuals aged 30‐50 years. It is usually found on the tongue, palatal complex (higher predilection), uvula, and gingiva. Some of these tumours have been linked to human papillomavirus (HPV) 6 and HPV 11, making them one of the most prevalent benign epithelial tumours [1]. It is unknown if all oral papillomas are caused by viral infection. The preferred course of treatment for these lesions is surgical excision, which can alternatively be accomplished with electrocautery, cold-steel excision, laser ablation, cryosurgery, or intralesional interferon injections [2].

It has been extensively documented how traditional medicine and complementary and alternative medicine (T/CAM) are becoming more and more popular among the general people. T/CAM is now frequently used by almost half of the population in several industrialised nations (United States, 42%; Australia, 48%; France, 49%; Canada, 70%), and is widely used in many developing nations (China, 40%; Chile, 71%; Colombia, 40%; up to 80% in African nations) [3].

Custard apple *(Annona squamosa)* leaves (ASL) have been investigated for their health advantages, which are attributed to a wide variety of phytochemicals. The biological effects of ASL extracts, including their hepatoprotective, anticancer, antidiabetic, antioxidant, antibacterial, anti-obesity, and lipid-lowering properties, have been investigated [4,5].

No publication mentioning the use of ASL for the management of oral squamous papilloma was discovered after an extensive search of scientific databases. This case presentation is an example of this novel observation.

## Case presentation

A 36‐year‐old male presented with a raised whitish-pink lesion on the left side of the palatal mucosa. According to the patient, the lesion's size had progressively grown over the past year without any obvious cause for aggravation or relief, and the patient had not taken any special measures to treat it.

**Figure 1:**
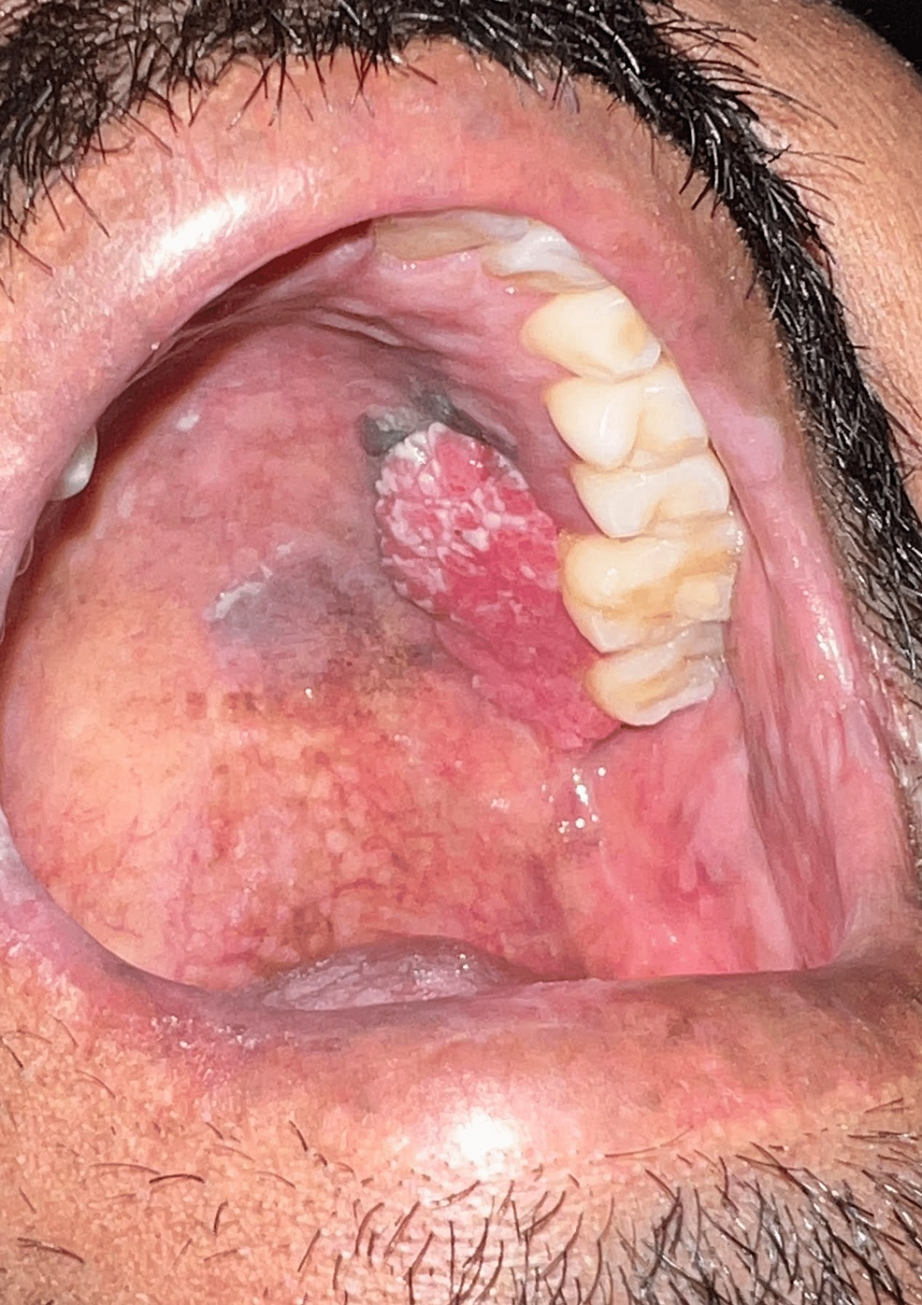
Oral squamous papilloma on the left maxillary palatal region

A firm, single, well-defined exophytic sessile growth with a rough verrucous surface was found on the intersection of the hard and soft palates, originating along the cervical margins of 27 and extending up to 24, roughly the size of 2.5 X 1.5 cms, according to an intraoral examination (Figure 1). There were no palpable lymph nodes. No accompanying pain, paresthesia, or discomfort during speech or deglutition existed for the patient. There were no other lesions on the body with a comparable appearance.

The patient was a known diabetic and was under management for it. No personal information on their usage of any substances, such as alcohol, tobacco, or other drugs was provided. The patient did not have a multi-partner/high-risk/unsafe sexual history. Upon a thorough haematological evaluation, all parameters aside from blood sugar levels were found to be within normal ranges. HCV, HIV, and hepatitis B surface antigen (HBsAg) viral indicators all tested negative. The provisional diagnosis of OSP was given on the basis of clinical information, and it was distinguished from other common benign and malignant diseases present in the oral cavity, such as fibromas, verruciform xanthomas, papillary hyperplasia, and condyloma acuminatum.

After receiving written informed consent, a biopsy was carried out under local anaesthesia, and tissue measuring 0.5 X 0.2 X 0.1 cm was sent in 10% formalin for histopathological analysis. The histopathological section showed squamous epithelium-lined fragments thrown into the shape of papillae and a central fibrovascular core with sparse lymphocytic infiltration and a few thin-walled blood vessels. Mild hyperkeratosis and inflammatory cell infiltrates were visible in the surface epithelium, although the surface maturation was intact. The area that was investigated revealed no dysplasia or cancer, providing the impression of a squamous papilloma, which was compatible with the provisional clinical diagnosis.

Electron microscope analysis reveals viral particles. In situ hybridization, immunohistochemistry, and polymerase chain reaction (PCR) procedures are the additional detection methods [6]. There was no viral genetic typing done. We decided that no additional testing was required because neither dysplasia nor neoplasia was visible.

The patient was slated for excision.

**Figure 2:**
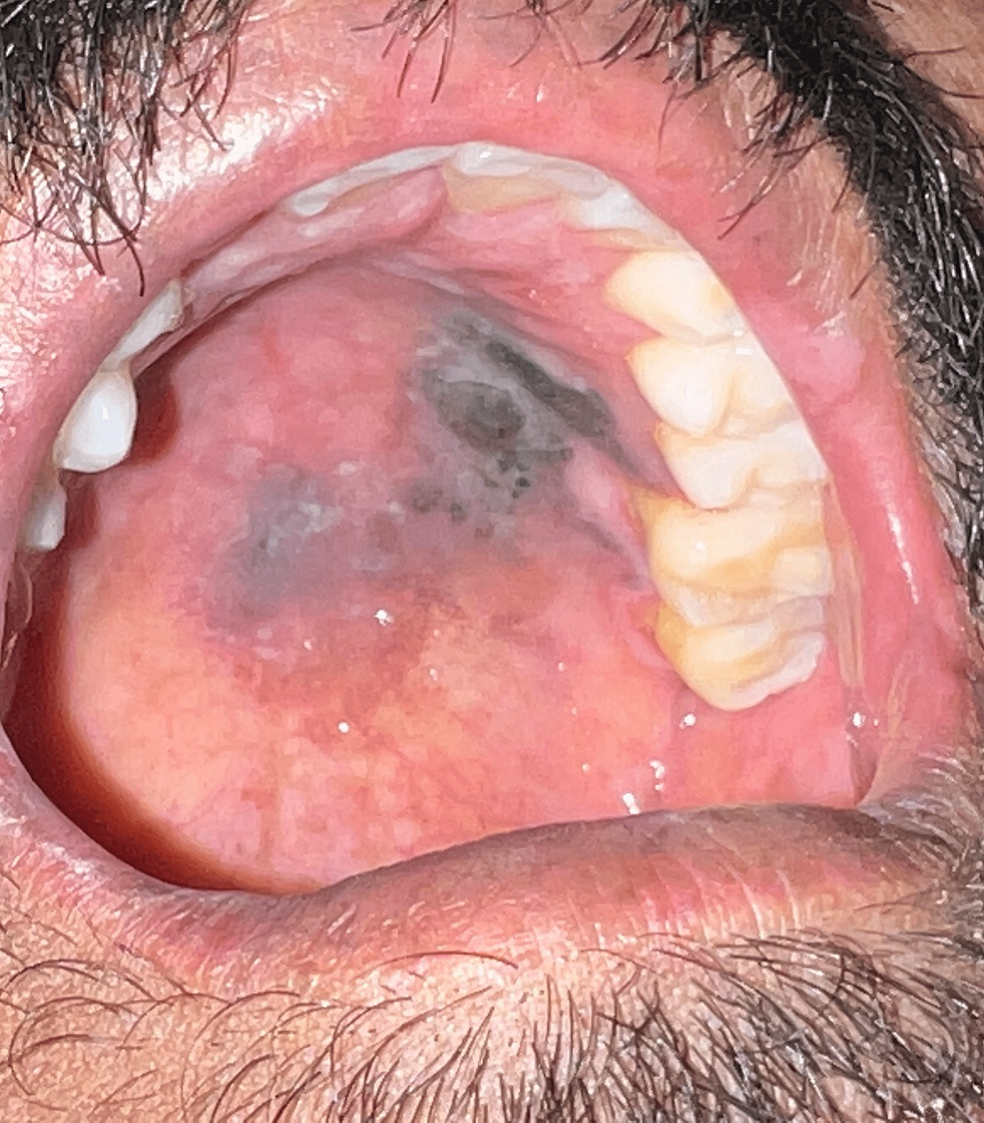
Healthy palatal tissue after self-prolapsed oral squamous papilloma

After a gap of one month, the patient returned to the department, and during examination, it was discovered that the squamous papilloma had entirely disappeared. Following an in-depth conversation with the patient, we concluded that neither any other medical professional had performed any surgical procedures nor had the patient used any medicines targeting the OSP. The only thing he did was that on the advice of a sibling, he utilized ASL. The lesion spontaneously prolapsed during the course of a few days after the patient chewed on a few ASLs and spit the remainder out. There were no obvious surgical scars nor any signs that a cauterization had occurred. This strengthened his claim (Figure 2).

Recurrence is uncommon, with the exception of people who are HIV-positive [7]. However, the patient was advised regular follow-ups to check for recurrence.

## Discussion

According to WHO, squamous cell papilloma is a benign hyperplastic exophytic localized proliferation with a verrucous or cauliflower-like morphology. Squamous cell papillomas may be pedunculated or sessile. The pedunculated lesions are composed of a cluster of finger-like fronds and may be white or mucosal in colour, depending on the degree of keratinisation [8]. It is a non-tender exophytic growth that resembles a lesion with a roughened, verrucous, or cauliflower-like surface and is composed of numerous tiny sharp or blunted finger-like projections. Depending on the level of keratinization, the colour may appear white or raspberry-like/pink. The size is typically tiny, with a diameter of a few millimetres to less than 1 cm, although occasionally, enormous growths up to 3 cm in diameter can be found [9]. It is made of a supporting core of connective tissue and a tiny quantity of benign epithelium. OSP was initially described as a “gingival wart” by Tomes in 1848 [6].

HPV 6 and HPV 11 may be linked to it since it is most usually detected on the tongue, palate, uvula, and gingiva. Up to 50% of oral papillomas have been found to contain the HPV 6 and 11 strains [6].

Papilloma is the fourth most common oral soft tissue lesion, accounting for 3-4% of all biopsied lesions [10]. Males are significantly more likely to develop it than females, and the White race is more likely to do so than other races [11]. Smoking, concurrent infections, nutritional deficits, and hormonal shifts all affect the likelihood of developing these lesions [7].

The first line of treatment is surgical removal, with a suggested clearance of 1 mm from the base to the depth of the submucosa. Other options include electrocauterization, cryosurgery, laser surgery, and interferon injections. Topical cidofovir in patients who are HIV positive needs to be explored further [12]. Salicylic acid and vitamin A have both been suggested as topical treatments [13]. 

It must be carefully removed from highly vascular areas while keeping other critical structures such as nerves and blood vessels in mind. OSPs normally do not turn malignant or recur after surgical removal [9].

Plants and the bioactive components these contain have been used for medicine since the dawn of humanity. Herbal medicines are a gift from nature to humans. The potential of natural products and phytochemicals produced from plants in the treatment of oral disorders has been documented by various writers in recent years [14]. A sizable fraction of the world's population uses traditional medicines to treat a range of illnesses. The term "home" or "folk" remedies is used to describe T/CAM in developing countries where it has been used for a long time both inside and outside the current healthcare system. By 2018, 124 WHO Member States had passed legislation governing herbal remedies, 109 had put national T/CAM laws or regulations forward, and 98 had created national T/CAM policies [15]. In addition to safety and efficacy concerns, regulation has also been a point of concern for WHO in past in the matter related to T/CAM [3]. Anthocyanins, ginseng, curcumin, lycopene, and artemisinin are a few of the possible substances that have demonstrated promise in the treatment of oral squamous cell carcinoma (OSCC) and other tumours [14].

The custard apple (*Annona squamosa*), of the family Annonaceae, is a popular tropical fruit known for its health benefits. The West Indies, South and Central America, Ecuador, Peru, Brazil, India, Mexico, the Bahamas, Bermuda, and Egypt all farm this significant tropical fruit [4].

The health advantages of ASL, which are attributable to a wide variety of phytochemicals, have been examined and are used as a folk remedy. These substances include phenol-based substances, such as proanthocyanidins, which are made up of 18 different phenolic substances. The majority of these are total phenolic compounds, which also include flavonoids, alkaloids, phenols, saponins, and tannins. The biological effects of ASL extracts, including their hepatoprotective, anticancer, antidiabetic, antioxidant, antibacterial, anti-obesity, and lipid-lowering properties, have been investigated [4,16]. The protective effects of polyphenolic compounds against a number of chronic diseases, including cancer, diabetes, cardiovascular, and neurological diseases, have been established by epidemiological studies [4].

In Japan, the herbal medicine keishibukuryogan-ka-yokuinin (KBGY) is used to treat different skin conditions, including viral warts. KBGY is a combination of keishibukuryogan, which is made up of five medicinal plants [17], and yokuinin (coix seeds extract) [18]. Yokinin may be antiviral while keishibukuryogan may be helpful for skin or oral mucosal remodelling, and the impact of KBGY on papillomas is therefore positive [19].

Studies have found that garlic (*Allium sativum*), which is frequently used in traditional and alternative medicine, has potent antiviral effects. Many different forms of warts, including verruca plana, are treated by pounding garlic into a paste and applying it topically; however, an inappropriate application might have unfavourable effects like the Koebner phenomenon [20].

Examples of ethical problems that may occur include difficulties with patient recruiting, randomization, and retention, as well as the identification of permissible placebo therapies. Furthermore, the ready availability of nutritional supplements and other complementary interventions on the free market considerably increases the potential of "cheating" by the control group should patients agree to randomization.

Despite these challenges, carefully thought-out clinical trials are still possible, including in-depth analyses of entire T/CAM systems. Observational studies provide a useful way to tackle particular types of issues, such as the assessment of occasional unfavourable occurrences. People who maintain that the evidence for T/CAM can legitimately be weaker than mainstream care will need to reevaluate their views.

Integrating T/CAM practitioners within settings of traditional medicine is essential. This was done in India by incorporating AYUSH (Ayurveda, Yoga and Naturopathy, Unani, Siddha and Homeopathy) clinics within standard conventional medical facilities. The National Health Policy 2017 has provided significant support for AYUSH's potential within a varied system of integrative healthcare [21]. Conventional healthcare practitioners need to learn as much as they can about T/CAM in order to make sure that their patients feel comfortable discussing their usage of T/CAM with them.

Natural dietary phytoconstituents will continue to be an intriguing and vibrant study subject in the coming years. Large-scale, meticulously supervised clinical trials are required to examine the efficacies, side effects, and safety of promising phytochemicals derived from plants. Comparisons of alternative and conventional medicine are necessary for the treatment of specific diseases. In addition to specific biomedical outcomes, comparative studies could assess the viability, affordability, and environmental impact.

Our report’s obvious limitation is that it only considers one specific example. But to believe that there is a seed of truth in a patient's words is a starting point.

## Conclusions

This report is unique because our search of scientific literature turned up no published works in which the use of ASL for OSP treatment was highlighted. In order to establish the repeatability of our novel observation with ASL on papillomas, well-drawn-out research with a larger sample size is required. Given their lower cost and less reliance on an expensive resource base, these food-based drugs may be more readily accepted. Reduced healing time or less wound contracture/scarring may also lessen patient anxiety. There is an urgent need for local health traditions to be globally revived in a more uniform way. The future is an amalgamation of all streams, and perhaps this will be decided by objective scientific analysis.
